# Advances in Citrus Flowering: A Review

**DOI:** 10.3389/fpls.2022.868831

**Published:** 2022-04-08

**Authors:** M. Agustí, C. Reig, A. Martínez-Fuentes, C. Mesejo

**Affiliations:** Instituto Agroforestal Mediterráneo, Universitat Politècnica de València, Valencia, Spain

**Keywords:** *Citrus*, flowering genes, floral induction, floral differentiation, carbohydrates, juvenility, plant hormones

## Abstract

*Citrus* are polycarpic and evergreen species that flower once in spring or several times a year depending on the genotype and the climatic conditions. Floral induction is triggered by low temperature and water-deficit stress and occurs 2–3 months before bud sprouting, whereas differentiation takes place at the same time as sprouting. The induced buds develop single flowers or determinate inflorescences, so that vegetative growth is required at the axillary buds to renew the polycarpic habit. The presence of fruits inhibits sprouting and flower induction from nearby axillary buds in the current season. In some species and cultivars, this results in low flowering intensity the following spring, thus giving rise to alternate bearing. A number of key flowering genes act in the leaf (*CiFT3*, *CcMADS19*, etc.) or in the bud (*CsLFY*, *CsTFL1*, etc.) to promote or inhibit both flowering time and reproductive meristem identity in response to these climatic factors, the fruit dominance, or the age of the plant (juvenility). The expression of some of these genes can be modified by gibberellin treatments, which reduce bud sprouting and flowering in adult trees, and constitute the main horticultural technique to control flowering in citrus. This review presents a comprehensive view of all aspects of the flowering process in citrus, converging the research published during the past half century, which focused on plant growth regulators and the nutritional source-sink relationships and guided research toward the study of gene transcription and plant transformation, and the advances made with the development of the tools of molecular biology published during the current century.

## Introduction

In *Citrus*, flowering time and intensity depend on the species, the tree age, and the climatic conditions. Flower load can reach up to 250,000 flowers per tree, although usually less than 1% becomes a mature fruit. Some species and cultivars flower once or more times (e.g., *Citrus limon*) a year (*regular bearing*). Other ones alternate years of profuse flowering and yield with years with few to no flowers (*alternate bearing*).

Prior to flower development, the buds must be activated by the interaction between exogenous and endogenous factors, which promotes the changes in their cells necessary for the formation of specific structures, such as inflorescences. This change from a vegetative meristem to an inflorescence or floral meristem is called *floral induction* (*flowering time*), and although it begins in the leaves with the synthesis of new proteins, it exerts the action in the buds, controlling the *meristem identity* and the phase transition from vegetative to reproductive development and flower formation (*floral differentiation*). These three phases, induction, differentiation, and *organogenesis*, are regulated differently and independently.

## The Flowering Process

In citrus, floral induction and floral differentiation take place 2–3 months before and at the time of bud release, respectively. At the beginning, in the transformation from vegetative meristem to flower bud, flattening of apex takes place and gives rise to the receptacle of the flower and the initiation of petals, stamens, and carpels (Lord and Eckard, 1985). When the bud is released from dormancy, flower primordia appear as emerging fingers of the meristem curving on themselves and giving rise to the flower bud.

**Figure 1:**
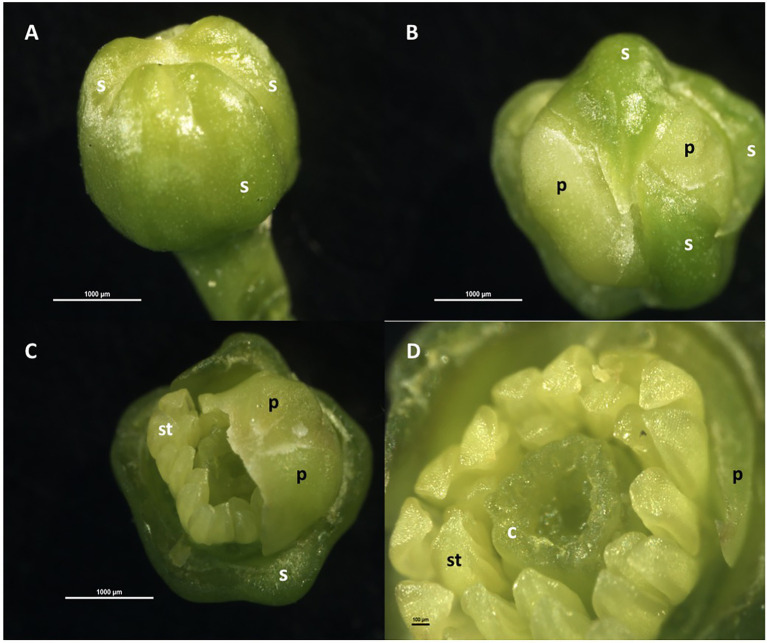
Floral organogenesis in *Citrus sinensis*. **(A)** Floral bud with sepals and petals beneath; **(B)** floral bud with two sepals removed, showing whorl of petal primordia; **(C)** floral bud with sepals and three petals removed, showing whorl of stamen primordia; **(D)** floral bud with sepals and petals removed, showing whorls of stamen primordia and carpels initiating. c: carpel, p: petal, s: sepal; st: stamen.

Floral organogenesis occurs in an acropetal manner, that is, from the most external to the most internal organ, so that each whorl is formed above and within the preceding one. Thus, the sepals are the first to form, followed by the petals, and later, internally and concentrically to them, the stamens that form a simple whorl; afterward, the pistil is formed in the innermost zone (Figure 1). The pistil has an ovary, with a whorl of 8–10 carpels containing 2–3 ovules each, and a single style with a papillated stigma. The nectariferous disc is located between the whorl of carpels and the stamens (Schneider, 1968; Lord and Eckard, 1985; Davenport, 1990; Martínez-Alcántara et al., 2015b). In the ovary, the papillary cells of the placenta, which form a continuum with the cells of the stylar canal, help the pollen tubes to reach the ovules (Distefano et al., 2011).

At the onset of flower development sepals completely cover the bud. Later, the petals begin to be visible, while inside, the anthers of the stamens reach the height of the stigma. The continuous growth of the petals pushes the sepals outward and outgrow them. At that time, the stigma protrudes from the anthers. Close to reaching their final size, the petals continue to overlap each other, forming a kind of globe that surrounds both the androecium, whose stamens have grown considerably, and the gynoecium. Subsequently, anthesis occurs, the petals are open, the anthers dehisce and the stigma is receptive to pollen (Schneider, 1968; Lord and Eckard, 1985; Davenport, 1990). The stigma receptivity in Clementine mandarins (*Citrus clementina*) and sweet oranges (*Citrus sinensis*) and the ovule receptivity in Satsuma mandarins (*Citrus unshiu*), determine the effective pollination period (Mesejo et al., 2007).

## Flower Distribution

After the winter rest period buds sprout and flower development begins in spring. The new shoots originate from the axillary buds located on the shoots of the previous year, and only occasionally dormant buds develop. Later, in summer and autumn, buds sprout again, but these new shoots do not develop flowers. Among the shoots of the previous year, the axillary buds of the autumn are the ones that sprout earlier in spring, in a greater proportion, and produce more nodes, followed by those of the summer shoots and these, in turn, by those of the previous spring (Agustí et al., 1982). Each node can develop one or more shoots at sprouting, in accordance with its number of buds. The percentage of nodes in which the buds remain dormant is higher in the older shoots (Guardiola et al., 1982). The significant contribution of summer shoots to return bloom was quantified for *Citrus reticulata* grown in the Mediterranean climate of California (Verreynne and Lovatt, 2009).

**Figure 2:**
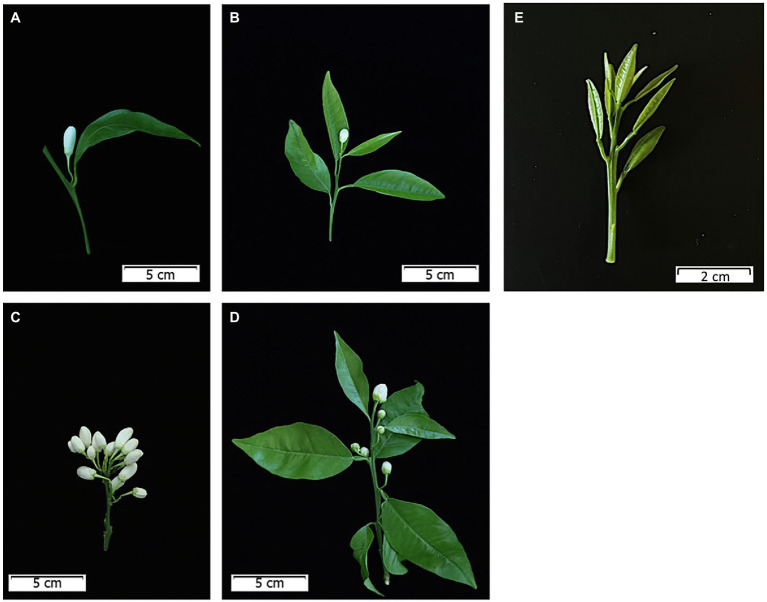
Type of shoots in *Citrus*. **(A)** Single-flowered leafless shoot; **(B)** single flowered leafy shoot; **(C)** multiflowered leafless shoot; **(D)** multiflowered leafy shoot; **(E)** vegetative shoot.

The flower buds develop determinate shoots, which can be mixed or generative shoots. In the former, the apical meristem of a leafy shoot develops into a terminal flower, and the axillary buds may, in turn, sprout, and when this occurs, these buds only bear flowers (Schneider, 1968; Lord and Eckard, 1985). In the generative shoots, the foliar primordia are inhibited at sprouting, giving rise to floral shoots without leaves. Hence, the presence or absence of leaves and flowers in combination gives rise to the five shoot types: (1) leafless inflorescences bearing one or (2) more flowers, (3) leafy inflorescences bearing one flower in a terminal position, and (4) many axillary flowers, and (5) vegetative shoots (Figure 2). The number of flowers and leaves depends on two factors: the number of primordia present in the bud, and the abscission or lack of development of some organs, mainly leaves (Davenport, 1990; Agustí and Primo-Millo, 2020). As a consequence of this, the transformation of some types of shoots into others occurs over time. This distribution of shoot types is identical for all cultivated species and varieties, although with quantitative differences. The most important difference occurs in the Satsuma mandarin and is based on the incompetence of the axillary buds of the newly developing shoots to sprout (Agustí and Primo-Millo, 2020). The development of leafless vs. leafy inflorescence was also correlated with the timing of sprouting and temperature during shoot development (Lovatt et al., 1984).

The fate of citrus buds, i.e., floral vs. vegetative, can be predicted based on the characteristics of the shoot on which the buds develop (named “floral” and “vegetative” mothers shoots; Lord and Eckard, 1987; Lovatt and Krueger, 2015). In addition, shoot with a terminal fruit always develops vegetative shoots (Muñoz-Fambuena et al., 2019). This provides a useful criteria that facilitates research on floral development.

## Endogenous Control of Flowering

### Genetic Factors

When a vegetative meristem receives the flower-inducing signal its genetic program is altered and becomes a reproductive meristem that gives rise to flowers. As mentioned above, this signal activates the floral induction and stablishes the onset of the transition from vegetative to reproductive development. Furthermore, the flower-inducing signal can be transmitted by grafting from an induced plant to a non-induced one, which indicates that the signal received by the meristem is stable, durable, and transmissible. In this sense, the leaves are essential for flowering since they receive the stimulus, produce the inductive signal and transmit it to the bud. In citrus, floral induction occurs during the autumn–winter rest period and the role of the leaves was demonstrated by defoliating trees in autumn and evaluating flowering in spring (Sánchez-Capuchino and Casanova, 1973).

Genetic pathways that promote flowering were defined in the model plant *Arabidopsis thaliana*. The timing of the onset of flowering depends on a few genes, the so-called floral pathway integrators. These genes integrate heterogeneous stimuli and induce the floral meristem identity genes which, when activated, initiate the ABC program of flower development and, hence, the transition from vegetative to reproductive meristem. The main genes responsible for integrating the inductive stimuli of flowering are *FLOWERING LOCUS T* (*FT*), *SUPPRESSOR OF OVEREXPRESSION OF CONSTANS 1* (*SOC1*), and *LEAFY* (*LFY*; Simpson and Dean, 2002). Among the floral meristem identity genes *APETALA1* (*AP1*) and *LEAFY* (*LFY*) play a key role, and the E-class *SEPALLATA* (*SEP*) genes, among others, regulate floral patterning (Pelaz et al., 2000). The *AtFT* gene encodes for the synthesis of the AtFT protein, which occurs in the phloem companion cells of the induced leaf and is transported in the sieve tubes to the meristem, where it forms a complex with the transcription factor *FLOWERING LOCUS D* (*FD*), so that the AtFD/AtFT heterodimer leads to floral initiation by activating the expression of *AtSOC1* and *AtAP1* floral identity gene (Abe et al., 2005; Wigge et al., 2005). The AtFT protein has been established to be the main, if not the only, component of florigen (Zeevaart, 2008). The *AtLFY* gene plays a key role during flower development, both for its temporal function and for floral identity (Blázquez et al., 1997). And *AtSOC1* integrates the environmental and autonomous signals that regulate flowering time (Samach et al., 2000). The *CONSTANS* (*CO*) gene is a flowering promoter in response to photoperiod; since the length of the day has little effect on the flowering of citrus, its role has been little studied in these species.

Coen and Meyerowitz (1991) defined three regions in the floral meristem, A, B and C, each coinciding with the domain of one of the three classes of flower homeotic genes. According to this model, it is the combination of these three classes of regulatory genes that determines the flower whorls. Those of class A (e.g., *APETALA2*) organize the first whorl with the formation of sepals; they, together with those of class B (e.g., *APETALA3* and *PISTILLATA*), organize the second whorl promoting the growth of the petals; those of class B and C in combination give rise to the development of stamens; and class C genes (e.g., *AGAMOUS*), alone, promote the formation of carpels in the center of the flower (Bowman et al., 1991; Irish, 2017).

In *C. unshiu*, three *FT* transcripts (based on EST databases) have been identified and characterized, *CiFT1* (AB027456), *CiFT2* (AB301934), and *CiFT3* (AB301935; Endo et al., 2005; Nishikawa et al., 2007); *CiFT1* and *CiFT2* transcripts are encoded by the same gene (Samach, 2013) and *CiFT3* correlated better to floral-inductive treatments than the other two (Nishikawa et al., 2007, 2009). Soares et al. (2020) developed two populations of *CcFT*-transgenic “Carrizo” citrange hybrid (*C. sinensis* x *P. trifoliata*) rootstock using *FT* citrus homologs cloned from *C. clementina*, *CcFT1* and *CcFT3* (MT707614 and MT602515, respectively, according to^1^); the transgenic *CcFT1* overexpressing lines did not flower, whereas those overexpressing *CcFT3* flowered. Accordingly, *CcFT3* induces flowering in citrus (Nishikawa et al., 2009; Soares et al., 2020). It is important to note that several of the papers published before 2021 show the transcript of *CiFT2* (Ciclev10012905m, according to^2^) as the factor controlling flowering time in citrus, due to the nomenclature adopted in the work published by Shalom et al. (2012, 2014). But Ciclev10012905m and MT602515 (*CcFT3*) encode for the same protein (100%), as well as AB301935 (*CiFT3*) from *C. unshiu*. *CiFT1* and *CcFT1* proteins share the 99.4% of their amino acid sequence. In this review, we maintain the nomenclature adopted by the authors of the cited papers.

The *FD-like* (*CcFD* and *CsFD*) genes (Zhang et al., 2021), *CsLFY*, *CsAP1* (Pillitteri et al., 2004a), two *SOC1* orthologs genes (*CsSL1* and *CsSL2*; Tan and Swain, 2007), and the *SEP* genes (*CiSEP1* and *CiSEP3*; Endo et al., 2006) have been also isolated and characterized in *Citrus* sp.

**Figure 3:**
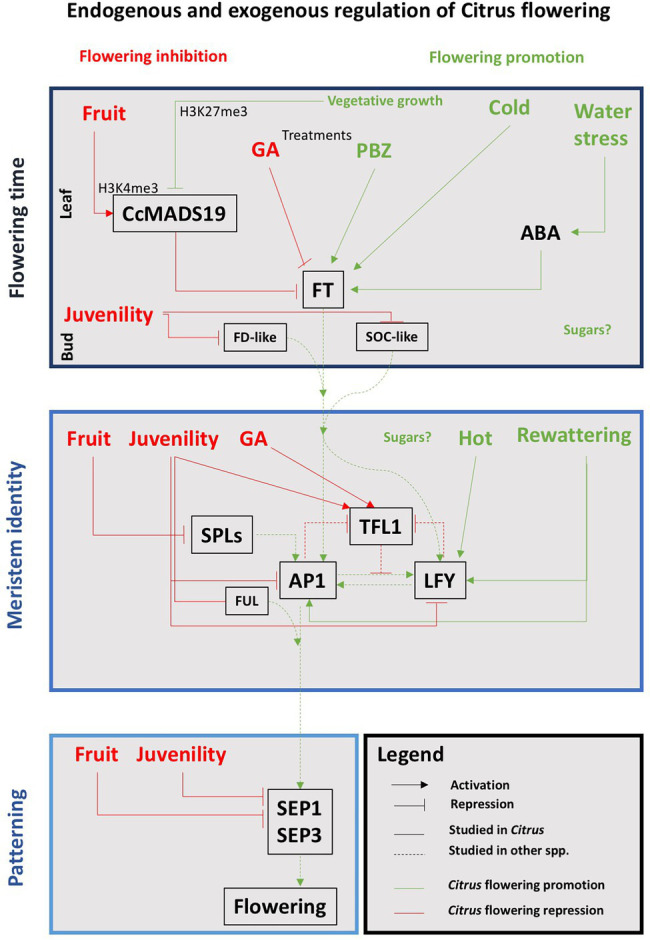
Schematic representation of the correlations between endogenous and exogenous factors and the main genetic pathways involved in the floral transition in *Citrus*. Red and green words and lines indicate that the interaction results in flowering inhibition or promotion, respectively. See text for details.

But there are also repressors that can reduce the expression of the flowering genes even under inductive exogenous conditions. Thus, *FLOWERING LOCUS C* (*FLC*) is a transcription factor that in Arabidopsis represses the expression of *AtFT* and *AtSOC1* (Helliwell et al., 2006). Its ortholog in *C. clementina* is *CcMADS19* (Hou et al., 2014), and also represses the expression of the *FT* gene in the leaves (Agustí et al., 2020). The *C. sinensis TERMINAL FLOWER1* (*CsTFL1*; Pillitteri et al., 2004b) is also a flowering repressor that represses *CcFD* transcription, keeping the meristem indeterminate and controlling its transition to flower (Zhu et al., 2020). The action of *AtTFL1* is also related to the repression of *AtLFY* and *AtAP1* activities in the dome of the meristem, and vice versa, *AtLFY* and *AtAP1* negatively regulate *AtTFL1* on the flanks of the meristem where the development of the flower begins (Ratcliffe et al., 1998; Samach et al., 2000). *CsTFL1* is expressed before the spring vegetative development ceases, but it shows low levels of transcription during the periods of floral bud induction and differentiation (Pillitteri et al., 2004b). *CENTRORADIALIS* (*CsCEN*), a *CsTFL1* homolog gene, is expressed in axillary meristems, where it interacts with *CsFD*, keeping them indeterminate. This pattern of *CsCEN* and *CsFD* co-expression and their interactions suggests that these genes act together to regulate axillary bud development (Zhang et al., 2021). Finally, *TEMPRANILLO1* (*TEM1*) is a *FT* repressor that regulates juvenility in olive (Sgamma et al., 2014) and also in citrus (Muñoz-Fambuena et al., 2019). Figure 3 shows a schematic representation of the interactions involved in the specification of floral meristems in *Citrus*.

### Environmental Factors

In temperate climates, low temperature during the autumn-winter rest period induces citrus flowering. For sweet oranges and some mandarins, floral induction occurs in autumn [(November–December in the Northern Hemisphere (NH)] (Guardiola et al., 1982), whereas for Satsuma mandarin it occurs later (January; Iwahori and Oohata, 1981; Guardiola et al., 1982). In contrast, in the hot areas of Tropical climate there are no significant changes in temperature throughout the year, so flower induction is not associated with low temperatures but a period of drought (see section “Water Stress Is Also a Factor to Induce Flowering in Citrus”), and the tree blooms several times a year; despite it, spring flowering is also the most intense. Consequently, low temperatures and water stress are the exogenous factors inducing flowering in *Citrus* (Figure 3).

#### Low Temperature Induces Flowering in *Citrus*

Indoor experiments showed that Satsuma mandarin trees grown at a temperature of 25°C for 7–9 months develop only vegetatively (Inoue and Harada, 1988). In contrast, trees grown at 15°C for at least 1.5 months develop flowers (Inoue, 1990), and this correlates positively with *CiFT3* expression (Nishikawa et al., 2007). The *CsLFY*, *CsAP1* and *CsTFL1* genes do not show changes in their expression associated with the 15°C-inductive period. This is consistent with their function as floral meristem identity genes since they increase their expression when the floral differentiation of the meristem is already microscopically visible (Nishikawa et al., 2007).

In the same way, under field conditions in the Mediterranean basin, the expression of *CiFT2* in the “Moncada” mandarin [*C. clementina* x (*C. unshiu* × *C. nobilis*)] starts when the minimum temperature drops below 15°C (Agustí et al., 2020), and the same occurs in the Satsuma mandarin in Japan (Nishikawa et al., 2007).

However, this temporal dependence on the genus *Citrus* is markedly different from other close genera, such as *Poncirus* and *Fortunella*, of the Rutaceae family. *Citrus* and *Fortunella* are evergreen species, whereas *Poncirus* is deciduous. *Fortunella* (Kumquats) trees bloom much later than those of *Citrus*, during summer and fall, and those of the genus *Poncirus*, although evocation takes place in summer, bloom early in spring (Hodgson, 1967). As for *Citrus*, in these genera, changes in *PtFT* gene expression correlate with the seasonal periodicity of flowering, however, whereas in *Citrus* species the *CiFT* expression increases in autumn–winter (Muñoz-Fambuena et al., 2011), in those of *Fortunella* and *Poncirus* it does in late spring–early summer, just before the floral initiation, so this must be their period of floral induction (Nishikawa et al., 2007, 2009; Nishikawa, 2013).

#### Water Stress Is Also a Factor to Induce Flowering in *Citrus*

As mentioned above, in the areas of Tropical climate, flowering occurs in response to rain after a period of drought. In Corpoica foothills, in Villavicencio (Meta), Colombia, latitude 1°–4° N, the average annual temperature is 25.5°C and the minimum never drops below 20°C, the average annual thermal amplitude is 6.9°C, and the average annual rainfall is around 3,000 mm. The latter is distributed mainly from April to November, with well-defined periods of high rainfall throughout the year, leaving dry periods of 2–3 months in between. Under these conditions, the “Valencia” sweet orange trees sprout continuously throughout the year (for a period of 10 months), but they do so with greater intensity during the rainy seasons and their sprouting diminishes with the arrival of the dry season. Trees also bloom several times a year and always in response to the water stress of a dry season (Orduz-Rodríguez and Garzón, 2013). Thus, in these areas, flower induction is associated with the water stress of the dry season (s).

This effect of water stress as a promoter of flower induction has been demonstrated in the sweet orange “Washington navel” even under non-inductive temperature conditions (Chica and Albrigo, 2013). The expression of the *CsFT3* gene in leaves of trees subjected to a period of 40–60 days of moderate water deficit (Ψ of – 2 MPa, measured in the leaf) increased in a similar way as it did with low temperature and, in addition, increased with the duration of stress. The floral identity genes, *CsLFY* and *CsAP1*, only were expressed when stress was broken with irrigation (Figure 3). And this effect was also similar to that obtained with low temperature in which flower differentiation only occurs after floral induction but not simultaneously, as occurs in many deciduous species. The consequence of the expression of these genes due to the action of a water deficit is the greater number of inflorescences and, therefore, of total flowers, compared to those well irrigated (Chica and Albrigo, 2013).

### Endogenous Factors

#### The Presence of Fruit as a Repressor of Flowering

In *Citrus*, the fruit inhibits flowering when it remains on the tree until the floral bud inductive period (November–December in the NH). Thus, in the Mediterranean basin, when the fruits are harvested before November, they hardly influence the following flowering, but if they are harvested later, flowering is partially inhibited, the more intensely the longer they remain on the tree. In such circumstances, the number of fruits also strongly influences the process, so that the greater the number of fruits, the fewer the number of flowers produced the following spring. Both factors, harvest time and number of fruits, have statistical interaction (Martínez-Fuentes, 2010). Therefore, a late harvest of a heavy crop load strongly depresses the following spring’s flowering, whereas an early harvest of a slight yield hardly affects flower formation.

The time of bud sensitivity to the fruit coincides with that of floral bud induction. In mandarin varieties highly sensitive to this effect (e.g., “Moncada”), the time-course of the *CiFT* expression in the leaves of trees with few fruits (OFF season) increases more than 10 times from September to December, but in trees with heavy fruit load (ON season) *CiFT* is strongly repressed from September to February (Muñoz-Fambuena et al., 2011). At the same time, the expression of the floral identity genes (*CsAP1* and *CsLFY*) in the buds is considerably reduced (Muñoz-Fambuena et al., 2012a; Shalom et al., 2012). Consequently, flowering is repressed.

**Figure 4:**
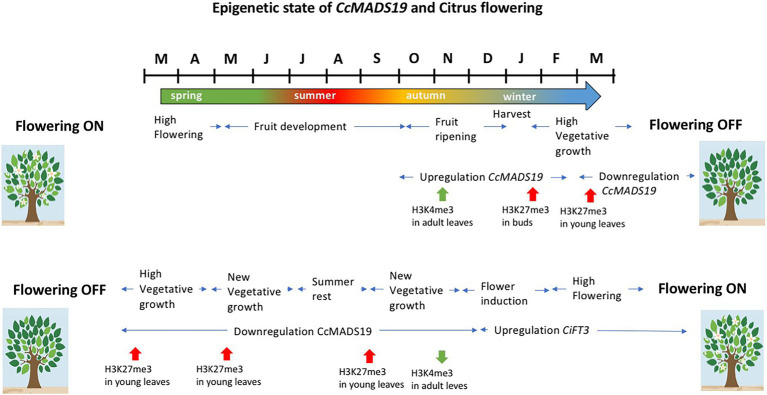
Schematic representation of the events that lead to flowering inhibition or promotion in alternate bearing *Citrus*, and the epigenetic state of *CcMADS19* in leaves and buds in relation to flowering inhibition and flowering resetting. See text for details.

The effect of the fruit is due to the epigenetic activation of *CcMADS19* in the adjacent mature leaf, but not in the buds, locally and temporarily repressing the expression of *CiFT2*. The *CcMADS19* active/repressed state is correlated with changes in histone methylation on the promoter of the *CcMADS19* locus: the active/repressed state in the leaves/buds correlates with enrichment of H3K4me3/H3k27me3 marks, respectively (Agustí et al., 2020). Since the axillary buds retain a silenced version of the flowering repressor gene (i.e., *CcMADS19* gene is enriched in repressive marks H3K27me3) that is mitotically transmitted to new vegetative shoots, the new leaves are able to induce flowering during the OFF season (the one with no flowers and fruits). This necessary reprogramming of the shoots allows flowering in the following season (Mesejo et al., 2021). Therefore, in citrus, flowering is necessarily preceded by sprouting, in order to re-establish the ability of young leaves to respond to the thermal/water deficit signals that induce flowering (Figure 4).

The intensity of alternate bearing varies among species and varieties. Thus, in some of them a large yield hardly affects the next flowering, whereas in other varieties fruits reduce flowering until almost completely nullifies it, even with a moderate crop load. This depends on the number of buds that are under the influence of the fruits, so that trees with many fruits are more sensitive than those with few fruits, even having a similar yield. Fruit reduce summer–autumn sprouting and, thus, the number of nodes that can bear flowers the following spring, and also spring sprouting, so that the reduction in flowering caused by the fruit is parallel to that of the bud sprouting (Verreynne and Lovatt, 2009; Martínez-Fuentes et al., 2010).

The decrease in yield, one out of every two or more years, is not the only problem of these kind of species/varieties since the commercial value of the fruit may be affected. The years of low production, fruits are usually large and have thick and rough skin, whereas those of large production are small-size fruits.

#### Tree Age. Juvenility

Citrus tree seedlings are unable to flower even under favorable environmental conditions. This is known as the *juvenile period* or *juvenility*, and precedes the adult or reproductive period in which the plant responds to inductive conditions (Albani and Coupland, 2010).

In *Citrus*, during the juvenile period, the meristems are dormant or develop vegetatively, and the stems grow exponentially, do not flower, develop several characteristic morphological structures (thorns) from the lateral meristems, and root better (Lord and Eckard, 1987; Davies and Albrigo, 1994). As the number of stems increases, the competition for nutrients among meristems increases as well, and a gradual loss of apical dominance and geotropic orientation appears, indicating the end of the juvenile period (Albani and Coupland, 2010). These meristems progressively acquire the temporary flowering program, and when they flower for the first time, they are capable of doing so forever. In *Citrus*, this period lasts between 5 and 20 years, depending on the species (Spiegel-Roy and Goldschmidt, 1996).

Studies on the genetic causes of this phenomenon began with the transformation of juvenile *Poncirus trifoliata* and citrange plants, in which the *AtFT* and *AtLFY* and *AtAP1* genes, respectively, were overexpressed. In both cases, flowering time was shortened from 7 to 10 years to 3–12 months (Peña et al., 2001; Endo et al., 2005; Sinn et al., 2021). Furthermore, transgenic grapefruit (*Citrus paradisi*) and sweet orange (*C. sinensis*) juvenile plants, ectopically expressing *CsAP1* or *CsLFY* under the control of the *Arabidopsis* stress-inducible promoter *AtRD29A*, flowered under non-inductive (warm temperature, well-watered) greenhouse conditions (Orbović et al., 2021).

The possibility that the *CiFT* gene is not expressed in the leaves or stems of the juvenile plants is, apparently, the logical choice. And this is the case in the stems of 4-months-old Satsuma plants exposed to 15°C for up to 2 months (Nishikawa, 2013). However, the time-course of its expression in leaves demonstrated that half-year-old “Carrizo” citrange plants expressed *CiFT* just 15 days after exposure to the same inductive conditions, and 1- and 3-year-old plants, which are still unable to flower, show a *CiFT* gene expression as high as that of 10-year-old plants, which flower normally (Muñoz-Fambuena et al., 2019). Accordingly, it can be concluded that the lack of *CiFT* expression in leaves cannot be, by itself, the main cause of juvenility (Nishikawa et al., 2007; Muñoz-Fambuena et al., 2019).

In the stems of non-flowering 4-month-old plants of the “Washington” navel sweet orange (cultivar CRC3306A, juvenile) the levels of *CsTFL1* RNAs are 13-fold more abundant than in those of the adult trees and there is a correlation between its expression and juvenility (Pillitteri et al., 2004b). In “Carrizo” citrange, results for 6-month-old trees agree with it, but its expression in the buds is downregulated in trees from 1 to 7 years old (Muñoz-Fambuena et al., 2019), indicating that *CsTFL1* is not the cause of the lack of flowering in juvenile trees older than 1 year old either. Furthermore, in the stems of the Satsuma mandarin the expression of this repressor is reduced to values as low as those of the adult plants 15 days after the exposure to the inductive temperature (Nishikawa et al., 2007). On the other hand, the expression of this gene, *CsTFL1*, has not being related to that of the *CsLFY* and *CsAP1* genes in the stems and buds until the plant becomes adult (Pillitteri et al., 2004a; Nishikawa et al., 2007; Muñoz-Fambuena et al., 2019). In fact, in adult trees and under field conditions, the expression of *CsTFL1* has only been detected in the meristems of the vegetative shoots and has been related to the vegetative growth, as shown with its paralog *CsCEN* maintaining vegetative axillary meristem indeterminate (see section “Genetic Factors”).

Therefore, another hypothesis is that citrus juvenility is mainly determined in the meristem. In the experiments with “Carrizo” citrange, the expression of the *CsSL1*, *CsAP1*, and *CiSEP1* genes increased with the tree age, whereas that of *CcFD* and *CsLFY* were only upregulated in the adult flowering tree (10-year-old) up to 8 and 20 times, respectively (Muñoz-Fambuena et al., 2019). This shows (1) the meristems of young trees progressively reach the stage of vegetative maturity marked by differences in the expression of genes involved in flowering, and (2) the key role of the *CcFD* and *CsLFY* genes in the transition from vegetative to reproductive meristem. The hypothesis is supported by the fact that meristems of not-flowering adult trees (due to high fruit load) are able to transcribe these flowering network genes but fail to achieve the transcription threshold required to flower, due to *CiFT2* repression (by the fruit; Muñoz-Fambuena et al., 2019).

Accordingly, the meristem progressively achieves the flowering time program, and when it flowers for the first time, the derived meristems are always able to transcribe their genes and, thus, bloom, unless something prevents it. When this occurs, it is because the leaf does not achieve the level of *CiFT2* transcription required to flower. Therefore, the genetic inhibition of flowering time in juvenile trees is determined in the meristem, which is unable to transcribe flowering time and flower patterning genes (Muñoz-Fambuena et al., 2019), whereas in adult trees it is determined in the leaf, where the *CiFT2* gene is repressed by fruit (Muñoz-Fambuena et al., 2011, 2019).

Finally, circular RNAs (circRNAs), micro-RNAs (miRNAs), and long non-coding RNAs (lncRNAs) have also been related with juvenility and flowering time (Liu et al., 2017; Wang et al., 2017; Zeng et al., 2018).

#### Carbohydrate Levels

Carbohydrate supply has been pointed out repeatedly as a factor for flowering (Goldschmidt et al., 1985; García-Luis et al., 1995). In alternate bearing cultivars, a depletion of carbohydrates in all tree organs occurs after a year of heavy crop load and no flowers are formed in the following season (Martínez-Alcántara et al., 2015a). In fact, defruiting at an early fruit development stage partially restores carbohydrates, allowing for the formation of some flowers (Goldschmidt and Golomb, 1982). Despite it, the regulatory role of carbohydrates in citrus flowering is still controversial. Thus, in the “Wilking” mandarin (Goldschmidt and Golomb, 1982), for example, the accumulation of starch in leaves, branches and roots during the low temperature period prior to flowering were quantified and related to flowering, whereas in the “Valencia” sweet orange (Martínez-Fuentes et al., 2010) and “Lisbon” lemon (Lovatt et al., 1988) subjected to an inductive thermal regime, not significant changes were found in starch and glucose in the leaves throughout the inductive period. Moreover, in the “Valencia” sweet orange and “Wilking” mandarin, no differences at the end of winter were found in their leaf content of ON and OFF trees (Lewis et al., 1964; Jones et al., 1974). Finally, in the tangelo “Minneola” subjected to different decreasing thermal regimes, the starch content of the leaves, branches and roots also did not correlate with the intensity of flowering, which was in strict correspondence with the decrease in temperatures (Goldschmidt et al., 1985).

This lack of relationship between carbohydrates and flowering was confirmed by performing treatments that reduce (gibberellins) and promote (girdling) flowering (see sections “Flowering Inhibition” and “Flowering Promotion”). In the “Shamouti” sweet orange, girdling branches in early-autumn increases the starch contents of leaves and twigs and flowering, but when, in addition, gibberellic acid (GA_3_) is applied at the time of floral bud induction, the increase in starch is maintained and flowering reduced counteracting the girdling effect (Goldschmidt et al., 1985). In fact, girdling branches in summer–early autumn mitigates biennial bearing by promoting flowering of alternating citrus cultivars (Agustí et al., 1992; Goren et al., 2004), even when no increases in the level of carbohydrates are detected in the leaves and shoots (García-Luis et al., 1995). Besides, the shading of Satsuma mandarin trees markedly reduces the content of soluble sugars and starch, as corresponds to its unfavorable effect on photosynthesis, but its effect on sprouting and flowering is negligible (García-Luis et al., 1995).

Accordingly, there is no evidence that the level of carbohydrates in leaves, branches and roots limits flower formation, but it may have an indirect effect (Lewis et al., 1964; Goldschmidt et al., 1985; García-Luis et al., 1995). Thus, the limitation of carbohydrates caused by the presence of the fruit reduces sprouting (Martínez-Alcántara et al., 2015a), and a minimum threshold level is required as energy input for floral differentiation (Goldschmidt, 1999). In fact, abundant flowering after an OFF year causes rapid mobilization of carbohydrate reserves (Monerri et al., 2011), as explained by ADP-glucose pyrophosphorylase and *α*-amylase activities (Nebauer et al., 2014).

#### The Mineral Nutrition Status. The Role of Nitrogen

In some cases, mineral element deficiencies cause a weakening of vegetative shoots that is usually accompanied by abundant flowering. In particular, trees with low levels of nitrogen in the leaves show abundant blooms and low vegetative vigor. These changes in flowering as a consequence of nutritional deficiencies suggest a non-specific response associated with the vegetative weakness of the tree. Consistent with this, excess nitrogen leads to strong vegetative development and reduces flowering (Agustí and Primo-Millo, 2020).

But nitrogen (N) metabolism has also been indirectly related to the induction of flowering, based on the fact that low temperatures or water stress increase the level of NH_3_-NH_4_^+^ in the leaves (Lovatt et al., 1988). In “Washington navel” sweet orange nitrogen levels increases with the duration of the period of low temperatures (8 h per day at 15–18°C and 16 h at 10–13°C) and has been positively correlated with the number of inflorescences and with the number of flowers per inflorescence. In “Frost Lisbon” lemon, moderate water stress (− 2 MPa) also increases the foliar concentration of NH_3_-NH_4_^+^ that positively correlates again with the number of flowers per tree. It has been proposed that this accumulation of ammonia might increase the biosynthesis of polyamines in the leaf, particularly spermidine, which could play a role in flower formation (Lovatt et al., 1988; Ali and Lovatt, 1995). However, in the bud it has been shown that the concentration of polyamines does not correlate with low temperature or with flowering (Arias, 1999).

Actually, this role has not been confirmed in field-grown citrus. In the “Hernandina” mandarin, the application of polyamines (arginine, putrescine, spermidine or spermine), at a concentration of 100 mg l^−1^ during the floral bud inductive period, did not alter the flowering intensity (Arias, 1999). Furthermore, in an experiment with alternating bearing “Moncada” mandarin, ON and OFF trees were fertilized in summer *via* soil, or some of their mature leaves individually sprayed in spring with a 5% solution of urea enriched with 10.2% ^15^N to trace the allocation of reserve nutrients and subsequent translocation from source to sink. There were not significant differences in N translocation from leaves to newly spring shoots, irrespective of fruit load; therefore, the N availability by the young shoots is not affected by fruit. Moreover, in the bark of the branches of fruitless trees the minimum concentration of N was reached in April, coinciding with the high demand for growing shoots, whereas in those of fruiting trees it occurred in June, coinciding with the high demand for developing fruits. This lack of coincidence indicates that the vegetative and reproductive organs do not compete for N. Therefore, N reserves do not explain the differences in flowering between both types of trees (Martínez-Alcántara et al., 2015a).

Finally, the proteomic analysis of leaves and buds of fruiting and fruitless trees reveal differences in protein synthesis related to amino acid and carbohydrate metabolism, and in oxidoreductase activity (Muñoz-Fambuena et al., 2013a,b).

#### Rootstock

Rootstock can also affect flowering. In Australia, “Valencia” orange grafted onto “Emperor” mandarin and sweet orange rootstocks was associated with relatively low alternation, whereas grafted onto “Troyer” citrange and *P. trifoliata* rootstocks resulted in a higher one, and “Carrizo” citrange rootstock had intermediate values (El-Zeftawi and Thornton, 1975, 1978). In the Mediterranean Basin, sour orange rootstock has long been related to heavy alternation (Monselise and Goldschmidt, 1982). In Japan, citrus hybrid seedlings grafted onto Shiikuwasha (*C. depressa* Hayata) rootstock accelerate flowering compared with *P. trifoliata* rootstock, which can be used to reduce the juvenile period (Mitani et al., 2008). Under Tropical climate rootstock also affects flowering; experiments conducted in Colombia showed that “Valencia” sweet orange increased flowering progressively when grafted onto *Citrus yuma*, Cleopatra mandarin (*Citrus reshni* Hort. *ex* Tan), *Citrus amblycarpa* and Citrumelo 4475 (*C. paradisi* × *P. trifoliata*; Holguín et al., 1992), and in Brazil “Frost Valencia” increased flowering when grafted onto “Rangpur” lime (*Citrus limonia* Osbeck) compared to Cleopatra mandarin (Vasconcelos et al., 2008).

A more in-depth study was developed by Bennici et al. (2021) who registered significantly higher flowering intensity in “Tarocco Scirè” sweet orange when grafted onto “C35” citrange than onto “Swingle citrumelo” rootstock. This effect paralleled *CiFT2* expression during the inductive period, regardless of the fruit load; the authors concluded that the effect of the interaction cultivar/rootstocks on flowering intensity is mediated by the upregulation of *CiFT2* gene.

Soares et al. (2020) developed transgenic “Carrizo” citrange rootstocks expressing *CcFT3* genes under the control of the phloem specific *SUCROSE SYNTHASE 2* (*AtSUC2*) promoter. The transgenic *AtSUC2-CcFT3* was capable of inducing precocious flowers in budded juvenile non-transgenic scions. Flower bud initiation was observed within 21 days after grafting buds from a 1-year-old juvenile “Valencia” seedling. The authors suggest that ectopic expression of *CcFT3* in phloem tissues of Carrizo citrange triggered the expression of several genes to mediate early flowering.

#### The Role of Plant Growth Regulators in Flower Induction

Gibberellins (GA) inhibit the flowering process in a large number of perennial species (Wilkie et al., 2008). This knowledge derives from their action when applied exogenously (see section “Flowering Inhibition”) since their endogenous role in flowering is still not well understood. Linked to its action, auxins (Ax) also seem to be related to the process of inhibition (García-Luis et al., 1986; Haim et al., 2021). Cytokinins (CK) and ethylene have been related with flowering too (Sanyal and Bangerth, 1998). The regulatory role of abscisic acid (ABA) in citrus seems to be contradictory; it has been linked to the inhibition of flowering by the fruit (Shalom et al., 2014) and also to the promotion of the *FT* gene (Li et al., 2017; Endo et al., 2018). Therefore, the endogenous role of the plant growth regulators in citrus flower induction remains unclear.

In fruit trees, GA are associated with shoot growth but their role in flowering is the opposite (Goldschmidt et al., 1997). Thus, in citrus, there is an antagonism between vegetative growth and flowering, so that the flowers arise from short generative shoots and the intensity of flowering is inversely related to the vegetative development (Martínez-Alcántara et al., 2015a). On the other hand, the content of GA in October–December (in the NH) is higher in the buds (“Valencia” sweet orange) and the leaves (Satsuma mandarin) of the fruit-bearing shoots that do not flower than the vegetative non-bearing shoots that bloom abundantly; later, up to February, the differences become very small (Jones et al., 1977; Koshita et al., 1999). These and previous results gave rise to propose that the endogenous GA produced by the fruit could be a factor controlling flowering in citrus (Monselise and Goldschmidt, 1982; Martínez-Fuentes et al., 2004). To this hypothesis contribute: (1) the moment in which the fruit inhibits flowering (Martínez-Fuentes et al., 2010) coincides, in general, with a reduction in the concentration of GA in the fruit and an increase in the bark tissue of the shoot (Kuraoka et al., 1977; Gambetta et al., 2012), (2) the peak in the GA foliar content (Koshita et al., 1999) coincides with the period of the greatest sensitivity to GA applied exogenously to reduce flowering (García-Luis et al., 1986), and (3) the low inductive temperatures are accompanied by a transitory reduction in the GA concentration in the buds, which is followed by floral differentiation (Muñoz-Fambuena, 2015).

However, this regulatory action of GA has been questioned (Monselise and Goldschmidt, 1982; Goldschmidt et al., 1997; Martínez-Fuentes et al., 2004, 2013) since, (1) although their content in the leaves (Koshita et al., 1999) and in the phloem fluid (Saidha et al., 1983) is higher in the shoots bearing fruits than in the vegetative ones, no differences have been found in GA bud content of trees with and without fruit during the dormant period (Jones et al., 1977); (2) the moment in which the GA concentrations change during flower initiation–differentiation is not precisely established; (3) it is not known whether there is a GA threshold level that allows flower initiation-differentiation; (4) the fruit counteracts the flowering promotion effect of paclobutrazol, a GA synthesis inhibitor (Martínez-Fuentes et al., 2013); and (5) the application of GA_3_ reduces flowering but does not alter the expression of the *CcMADS19* gene in the leaf, as it does the fruit.

The origin of the endogenous GA found in the buds has been suggested to be the seeds (Hoad, 1978) and/or the pericarp of the fruit (Kuraoka et al., 1977; Gambetta et al., 2012). This hypothesis is supported by: (1) the high concentration of GA in the seeds of apple (Hedden et al., 1993), pears (Griggs et al., 1970), and also in citrus (Monselise and Goldschmidt, 1982; Bermejo et al., 2016), (2) seeded fruits inhibit flowering induction for the next season in a higher proportion than seedless ones (Monselise and Goldschmidt, 1982), (3) seeded varieties contain higher GA contents than parthenocarpic ones (Bermejo et al., 2016) and are more prone to alternate bearing. In contrast, there is no evidence that the destination of GA is the lateral or terminal buds or that the transported amount is sufficient. Furthermore, the gibberellin precursor GA_12_ that acts as a long-distance growth signal (Regnault et al., 2015) has not been found in citrus buds during the inductive period.

Due to these doubts, an alternative signal pathway has been proposed: GA would be the first messengers present at the sprout apex where they would stimulate indole-3-acetic acid (IAA) synthesis, and the polar Ax transport would act as a second messenger that would be the true transported signal capable of inhibiting flowering (see Bangerth, 2009). In *Citrus*, ON-crop buds have higher levels of IAA than OFF-crop buds (Shalom et al., 2014), i.e., fruit causes a strong polar Ax transport that is reduced by fruit removal, thus allowing Ax release from the bud; this suggests that the fruit might generate an Ax signal in the bud and apical meristem that would interfere with floral induction (Haim et al., 2021).

Regarding to the ABA, the expression of the *CcNCED3* gene that encodes for its synthesis presents higher expression in the buds of vegetative shoots than in those of fruit-bearing shoots, but the concentration is lower in the former (Jones et al., 1976; Shalom et al., 2014). A possible explanation for this apparent contradiction is that the synthesis of ABA not only occurs in the bud itself, but is also external to it and dependent on the presence of the fruit. This high level of ABA in the fruit-bearing shoots could reflect the stress imposed by the presence of the fruit in the shoot, or the fruit overload on the tree, as shown by Muñoz-Fambuena et al. (2013a,b), and might explain its inhibiting effect on sprouting and flowering when applied locally to the buds of Satsuma mandarin at the floral inductive period (García-Luis et al., 1986). Notwithstanding, it is not known if ABA inhibits flowering in alternate bearing varieties. Moreover, application of ABA to potted Satsuma mandarin trees correlates with a transient accumulation of *CiFT3*, and endogenous ABA accumulates in the shoots as a response to a floral inductive period at 15°C and under field conditions; in both cases, ABA content correlated with flowering intensity (Li et al., 2017; Endo et al., 2018). However, if flowering induced by water stress is mediated by ABA accumulation still remains unclear. Despite this, whether ABA plays a role in controlling flowering or simply keeping the bud in a dormant state has not yet been clarified. In summary, further studies to elucidate the role of ABA on flowering in citrus are needed.

Ethylene has also been proposed as a promoter of flowering, but not in citrus. However, since it inhibits the polar transport of Ax, its mode of action should be indirect and could be similar to that of TIBA, an inhibitor of IAA transport (Sanyal and Bangerth, 1998). In fact, not only ethylene, but most GA biosynthesis inhibitors also reduce, to some extent, Ax export from fruit (Ebert and Bangerth, 1981; Callejas and Bangerth, 1998) and growing shoot tips (Rahim et al., 2011; Burondkar et al., 2016), and some of them promote flowering (Martínez-Fuentes et al., 2013). Ethylene could act, therefore, by repressing the inhibitory factor of floral induction, that is, the polar transport of Ax.

Assuming that ethylene does not act directly on floral induction, only CK remain as the plant hormones stimulating flowering. It has been shown in several woody fruit tree species that CK are involved in flowering (Corbesier et al., 2003), both due to the endogenous content and floral induction relationship (Hendry et al., 1982; Chen, 1991; Hegele et al., 2006), and to the response to CK treatments (Ramírez, 2000) or techniques that promote their synthesis (pruning roots, arching or bending shoots, …; Sanyal and Bangerth, 1998).

In both annual and perennial plants, it has been shown that the application of benzyladenine can replace the environmental factor that induces flowering in a concentration-dependent manner (Srinivasan and Mullins, 1979; Bernier et al., 1988). Furthermore, “Eureka” lemon (Tisserat et al., 1990) and Satsuma mandarin (García-Luis et al., 1989) lateral buds cultured *in vitro* at inductive temperature (14°–20°C) flower when the solid medium is supplemented with sucrose and CK. Likewise, *in vitro* flowering in embryogenic cultures of “Kinnow” mandarin is increased when the medium is supplemented with kinetin (2 mg l^−1^; Singh et al., 2006). These experiments show that an optimal concentration of CK that stimulate meristematic capacity is required in the bud to flower (Lifeng et al., 1987). Too low CK activity, possibly as a result of an IAA inhibitory effect (see above), generally results in bud dormancy, and too high activity in an excessive mitotic capacity of the meristem that can result in a new vegetative sprouting. The action of these treatments can be explained by an Ax/CK interaction, so that the inhibition of Ax transport could be as important as its concentration in reducing the CK concentration, as has been demonstrated in annual transgenic plants (Muday and DeLong, 2001).

A question to be clarified is the origin of the CK that accumulate in the meristem during the floral bud induction. At first, it was accepted that their synthesis was mainly accomplished in the roots (Skene, 1968) and seeds (Hahn et al., 1974); nowadays it is known that they are also synthesized in the buds and their vicinity, contributing to the dormancy release (Chen et al., 1997; Bangerth, 2006; Shimizu-Sato et al., 2009).

Cytokinin have been also related with juvenility. Buds of juvenile “Pickstone Valencia” sweet orange trees accumulate higher amounts of CK than those of adult trees prior to breaking dormancy, resulting in a higher growth of juvenile trees (Hendry et al., 1982).

In summary, GA, Ax, and CK have been shown to be closely related to floral induction in *Citrus* species. And it is logical since a significant function of these hormones is to detect environmental and endogenous signals and integrate them into a single signal, inhibitory or stimulant, capable of influencing flower induction in a quantitative way. See El-Otmani et al. (1995) for further information.

#### The Role of Plant Growth Regulators in Floral Organogenesis

In floral organogenesis, a distinction must be made between the activity of the inflorescence meristem (IM), which generates the lateral organs (flowers), and that of the floral meristem (FM), which subsequently generates the floral organs. The FMs produce organs in whorls, whereas the IM and the apical meristem of the vegetative shoots (SAM) follow the phyllotaxis pattern. The developmental sequence of an inflorescence follows the transformation of the vegetative SAM into an IM and this one, in turn, into a FM. When this process is completed, a terminal flower appears and the inflorescence is named determinate, but if the *AtTFL1* gene acts repressing the FM identity genes at the distal part of the SAM, the IM identity is maintained and the inflorescence is named indeterminate (see Bull-Hereñu and Claßen-Bockhoff, 2011). In *Citrus*, the IM show an imbalance in favor of the differentiation of the FM, and consequently the floral organs, instead of the maintenance of the meristematic activity in the IM, giving rise to shoots of determined development, which result in the formation of flowers. But this is not what happens in the IM, and they can develop determined shoots, which differentiate a terminal flower, or indeterminate shoots that ensure its polycarpic capacity.

After the initiation of the FM, the outgrowth of the flower with the correct patterning is under the regulation of a considerable number of genes under hormonal control, with Ax, GA, and CK playing a central role.

The role of Ax in lateral organ initiation is well known. Thus, a high and transient flow of IAA in the vegetative meristem, determined by the polar orientation of the PIN protein, regulates lateral bud initiation (Reinhardt et al., 2000). In the same way, the polar Ax transport and a correct gradient distribution in the FM, also regulates the initiation of flower organs. In the primordium of the gynoecium, high levels of Ax at the apex determine the formation of the style and stigma, whereas in the middle zone that of the ovary, and in the basal zone they promote the differentiation of the gynophore (not in the case of citrus; Nemhauser et al., 2000). These responses to auxin are regulated by the *AUXIN RESPONSE FACTOR*S (*ARFs*) that are also involved in the flower organs formation (Finet et al., 2010). However, they are ineffective in regulating Ax redistribution between flowers, so that the local Ax biosynthesis in the meristem is considered a key factor in controlling flower organs development (Chandler, 2011). This gene family has been identified and isolated in *Citrus* in which regulates floral organs developmental timing (*CiARF3/4*) and flower development (*CiARF5/6/7/8/10/19*; Li et al., 2015). Auxins are also necessary in the stamens regulating pollen maturation and anther dehiscence (Cecchetti et al., 2008).

Gibberellin also play a role in flower patterning, although little is known in citrus. Studies with GA synthesis mutants of different annual species have shown that these hormones regulate the transcription of the floral identity genes *LFY* (Blázquez et al., 1998; Yu et al., 2004) and *AP1* (Yamaguchi et al., 2014), among others, and *LFY* reduces the GA levels during flower formation (Yamaguchi et al., 2014), all which indirectly influences flower fertility, petals growth, and the development of stamens (Jacobsen and Olszewski, 1991; Wilson et al., 1992). These effects are repressed by DELLA proteins (Cheng et al., 2004). In citrus, exogenous GA reduce expression of *SEP3*, *AP1*, *AP2*, *PI*, and other floral–organ–identity genes (Goldberg-Moeller et al., 2013; Tang and Lovatt, 2019). Besides, *CcGA20ox* and *CcGA3ox* genes regulate cell division in the ovary (Mesejo et al., 2016).

CK affect the expression of the flower identity and flower development genes and the activity of the FM, either directly, or through the CK/Ax ratio (Chandler, 2011). In the FM, CK increases the number of flowers, modifies flower development and promotes the elongation of the inflorescences and the FM (Li et al., 2010). However, the role of CK in *Citrus* floral organogenesis is still unknown.

## Horticultural Control of Flowering

In *Citrus*, yield and fruit quality largely depend on flowering intensity, so that in many cases its control is of great importance to improve the harvest (Agustí et al., 1982). Flowering control is understood in a broad sense, and depending on the variety it will require flowering inhibition or promotion. For example, varieties that bloom profusely need flowering reduction to improve fruit set and harvest, whereas alternate bearing cultivars require treatments to increase flowering after a year of heavy fruit yield.

### Flowering Inhibition

In some parthenocarpic citrus varieties (e.g., “Navelate” and “Powell” navel sweet orange, among others), low yield is a consequence of high flowering intensity (it is common to find more than 100,000 flowers tree^−1^). In these cases, 90–99% of the flowers abscise, and the more intense the flowering, the sooner they abscise (Agustí et al., 1982). This relationship among the flowering intensity, the number of abscised flowers, and the timing of abscission is explained in terms of competition among developing flowers. Hence, the greater the intensity of flowering the greater the competition and depletion of carbohydrates reserves, it resulting in low ovary weight and high final abscission, so that the fruit set is frequently less than 1% (Agustí et al., 1982). This low number of fruits is, in turn, the cause of the large flowering intensity the following year, thus stablishing continuous cycles of high flowering–low harvest that must be interrupted.

Since Monselise and Halevy (1964) reported that GA_3_ inhibits flowering when applied at the floral bud inductive period, the application of this plant hormone has become a very useful technique to solve this problem see reviews by Harty and van Staden (1988) and El-Otmani et al. (2000). Treatments at the time of bud sprouting (January–February, in the NH) also reduce flowering significantly by interfering floral bud differentiation (Guardiola et al., 1982). Properly timed applications of GA_3_ at a concentration between 25 and 100 mg l^−1^, (1) significantly reduce the number of buds that sprout, (2) revert floral buds to vegetative shoots (Guardiola et al., 1982; García-Luis et al., 1986), (3) significantly reduce the number of flowers per tree, up to 45–75%, depending on the cultivar and treatment conditions (Table 1), and (4) directly increase fruit set (Agustí et al., 1982). This increase in the number of fruits per tree becomes, in turn, an effective control of flowering for the following season. Despite this depressing effect of GA_3_ on flowering, the number of flowers and leaves per shoot is not altered. Treatment has been effective in sweet orange, Satsuma and Clementine mandarins, and hybrids. Importantly, this reduction of flowering also increases the effectiveness of specific techniques to increase fruit set, such as the application of GA_3_ at petal fall (Agustí et al., 1982) or girdling branches during the physiological fruitlet abscission (Agustí et al., 2020). Suitable concentrations for practical use are 25 mg l^−1^ for sweet orange and 10 mg l^−1^ for Clementine mandarin applied from mid-November to mid-December in Spain (Guardiola et al., 1977; Agustí et al., 1981), 100 mg l^−1^ for Satsuma mandarin applied during late January in Japan (Iwahori and Oohata, 1981) or 25 mg l^−1^ applied in mid-June in New Zealand (El-Otmani et al., 2000), and 35 mg l^−1^ for “Tahiti” lime applied in mid-December in Florida-USA (Davenport, 1983).

**Table 1:** Current uses of gibberellic acid (GA_3_) to reduce flowering in *Citrus*.

*Citrus* species and cv.	% inhibition	[GA_3_]	Reference
Sweet orange ‘Shamouti’	76	200 mg L^−1^ × 3	Monselise and Halevy, 1964
	92	200 mg L^−1^	
Sweet orange ‘Shamouti’	60	0.075 μg/bud	Goldschmidt and Monselise, 1972
Sweet orange ‘Valencia Late’	54.5	50*3 ng L^−1^	Moss, 1970
			Moss and Bellamy, 1973
‘Navel’ sweet oranges	76.2	100 mg L^−1^	Guardiola et al., 1977
	48	100 mg L^−1^	
	27	50 mg L^−1^	
	30	25 mg L^−1^	
	45	25 mg L^−1^	
	94	50 mg L^−1^ × 8	Tang and Lovatt, 2019
Satsuma mandarin	54	0.03 mM	García-Luis et al., 1986
‘Nova’ mandarin	30	40 mg L^−1^	Gravina, 2007
‘Montrenegrina’ mandarin	60	40 mg L^−1^	Gravina, 2007
Tangor ‘Ortanique’	35%	40 mg L^−1^	Espino et al., 2005
Clementine mandarin	12.4	20 mg L^−1^	Deidda and Agabbio, 1977
Tangor ‘Ellendale’	39.4	20–40 mg L^−1^	
	45.2	20 mg L^−1^	Gravina et al., 1997
Tangor ‘Ellendale’	45.7	75 mg L^−1^	Arias, 1999
Sweet orange ‘Salustiana’	55.6	75 mg L^−1^	Arias, 1999
	72	40 mg L^−1^	Muñoz-Fambuena et al., 2012b
Clementina m., cv. ‘Hernandina’	70.1	50 mg L^−1^	Martínez-Fuentes et al., 2004
Clementina m., cv. ‘Marisol’	14	50 mg L^−1^	
Clementina m., cv. ‘Orogrande’	38.1	50 mg L^−1^	
‘Orri’ mandarin	75	150 mg L^−1^ × 4	Goldberg-Moeller et al., 2013

The GA_3_ effect inhibiting flowering has been related to the repression of the genes *CiFT3*, in the leaf, and *CsAP1, CcPI, CcSEP3*, and *CsAP2* in the bud (Muñoz-Fambuena et al., 2012b; Goldberg-Moeller et al., 2013; Tang and Lovatt, 2019), thus explaining its action on flower induction, differentiation, and organogenesis. The expression of *CcMADS19* in the leaf is not modified by GA_3_, and neither is that of *FLC-like* in the bud (Goldberg-Moeller et al., 2013), which suggest a different mechanism to that of the fruit in the inhibition of flowering (see “The presence of fruit as a repressor of flowering”). Interestingly, in *Arabidopsis* the application of GA_3_ reduces the accumulation of DELLA proteins and they are necessary for *AP1* expression (Yamaguchi et al., 2014), so this might be the way of action of GA_3_ inhibiting flowering.

Auxins (2,4-D) applied in mid-November to mid-December at 12 mg l^−1^ also reduced flowering by 30%, approximately, in sweet orange in Spain (Guardiola et al., 1977). Higher concentration (18 mg l^−1^) had not an additional effect and lower (7.5 mg l^−1^) did not inhibited flowering (García-Luis et al., 1986).

No practical uses of cytokinin to reduce flowering in citrus have been reported.

### Flowering Promotion

Given that GA reduce flowering, their biosynthesis inhibitors were used to promote flowering (Monselise et al., 1966). Indeed, the application of growth retardants, such as B-nine, cycocel, benzothiaxole, or paclobutrazol, during the floral bud inductive period, both to the canopy or to the soil, increases the number of flowers (Monselise et al., 1966). Among them, the most widely used is paclobutrazol, which applied at a concentration of 1–2 g l^−1^ to OFF or medium yield trees increases the number of generative shoots and reduces that of vegetative shoots, thus increasing the number of flowers per tree (Delgado et al., 1986; Harty and Van Staden, 1988; Greenberg et al., 1993; Muñoz-Fambuena et al., 2012b; Martínez-Fuentes et al., 2013). The treatment does not modify the number of leaves per shoot but increases the number of flowers per inflorescence (Muñoz-Fambuena et al., 2012b). Unlike GA, paclobutrazol increases *CiFT2* expression in the leaves when applied during the floral bud inductive period (Muñoz-Fambuena et al., 2012b); however, the presence of a high number of fruits (ON trees) cancels its effect on flowering promotion, so this type of substance seems not to be effective to control alternate bearing (Martínez-Fuentes et al., 2013).

Branch ringing, or girdling, has also been used to promote flowering (Furr and Arsmtrong, 1956; Erner, 1988; Agustí et al., 1992). Ringing carried out in summer, between the end of July and the beginning of August (NH), promotes the development of vegetative shoots in early-fall and the leaves of the new shoots reach the flower induction period (November–December) mature enough to receive the inductive signal, and trees bloom in the forthcoming spring. But, again, for a high number of fruits per tree the effect is limited (Goldschmidt et al., 1985), since growing fruits prevent bud sprouting in summer and fall (Martínez-Alcántara et al., 2015a). Thus, another method based in the promotion of vegetative growth in the ON season to mitigate alternate bearing was developed with directed mechanical pruning. The method allows the formation of new buds that reach floral inductive period ready to be induced and flower in the next season. Results showed an increased cumulative yield by 25% with regard to unpruned trees during the 4 years of the trial (Mesejo et al., 2020). Finally, regulated water deficit is used in Dry climates (e.g., west Perú) to promote and advance flowering time after fruit harvest, and to induce a summer bloom (*forzatura*) to produce out of season *verdelli* lemons in the following summer in Mediterranean climate (Sicily, Italy; Barbera et al., 1985), and limes in Mexico.

## Conclusion

Understanding of flowering control in *Citrus* species has made great progress during recent years, particularly in the genetic control of the floral bud induction and differentiation processes. The role of the environmental factors, such as temperature and water stress, upregulating *CiFT3* gene expression makes it possible to understand the differences in the time and intensity of flowering between the different growing areas and climates. The understanding of endogenous factors inhibiting flowering, such as tree age (juvenility) and the effect of fruit in adult trees, has also been improved. The genetic inhibition of flowering in juvenile trees is determined in the meristem, which is unable to transcribe flowering time and flower patterning genes, whereas in adult trees it is determined in the leaf, where the *CiFT3* gene is repressed by fruit. Flowering time can be advanced in transgenic juvenile trees constitutively expressing *CiFT3*, *CsLFY* and *CsAP1*. However, the inhibitory effect of the fruit on flowering cannot be avoided in adult trees. The fruit induces the epigenetic activation of the *CcMADS19* gene (*FLC Citrus* ortholog) in the leaf which correlates with *CiFT3* repression. And the new vegetative shoots present a silenced version of the *CcMADS19* gene, this allowing flowering in the following season. The endogenous signal that produces the fruit to activate the flowering repression program still remains elusive, but the most recent research points to an hormonal signal which may be from gibberellins and auxins directly regulating at the meristem level. This hormonal hypothesis is compatible with the classical nutritional hypothesis, which assumes that depletion of carbohydrates by the fruit participates in the flowering inhibitory signal.

Finally, the most remarkable results obtained with plant growth regulators, girdling and pruning, to inhibit or promote flowering are presented to provide techniques capable to control flowering intensity. Some of them have been proved to modify the flowering genes expression, although with quantitative differences between experiments which confirms the horticultural limitations to control flowering in *Citrus*.

## Author Contributions

All authors listed have made a substantial, direct, and intellectual contribution to the work and approved it for publication.

## Conflict of Interest

The authors declare that the research was conducted in the absence of any commercial or financial relationships that could be construed as a potential conflict of interest.

## Publisher’s Note

All claims expressed in this article are solely those of the authors and do not necessarily represent those of their affiliated organizations, or those of the publisher, the editors and the reviewers. Any product that may be evaluated in this article, or claim that may be made by its manufacturer, is not guaranteed or endorsed by the publisher.

## Footnotes

^1^ https://www.ncbi.nlm.nih.gov/

^2^ https://phytozome-next.jgi.doe.gov/
